# Chemesthetic Perception in Extra Virgin Olive Oil and Olive Ripening Stage: A Sensory Perspective

**DOI:** 10.3390/foods15030519

**Published:** 2026-02-02

**Authors:** Sofia Panzani, Francesca Venturi, Isabella Taglieri, Giuseppe Ferroni, Chiara Sanmartin

**Affiliations:** 1 Department of Agriculture, Food and Environment, University of Pisa, Via del Borghetto 80, I-56124 Pisa, Italy; sofia.panzani@phd.unipi.it (S.P.); francesca.venturi@unipi.it (F.V.); giuseppe.ferroni@unipi.it (G.F.); 2 Interdepartmental Research Centre “Nutraceuticals and Food for Health”, University of Pisa, Via del Borghetto 80, I-56124 Pisa, Italy

**Keywords:** chemesthesis, olive maturity, sensory analysis, oleocanthal, oleacin, TRP channels, pungency, phenolic compounds, sensory perception

## Abstract

This review focuses on chemesthetic perception (i.e., pungency, tingling, and astringency) in extra virgin olive oil (EVOO), with particular attention to the sensory mechanisms underlying trigeminal stimulation elicited by phenolic secoiridoids, considering olive-fruit ripening as a key modulating factor. The chemesthetic profile represents one of the most distinctive sensory features of EVOO and is primarily associated with phenolic secoiridoids derivatives, formed through enzymatic transformations of ligstroside and oleuropein. Generally, a progressive decrease in chemesthetic potential is observed during ripening, due to the reductions in total phenols, *o*-diphenols, and secoiridoids. Among these compounds, secoiridoid derivatives, most notably oleocanthal and oleacin, elicit chemesthetic sensations and represent some of the most biologically active EVOO phenolic constituents. In this context, chemesthetic perception may work as a sensory marker of phenolic richness and nutraceutical value, linking sensory science with olive ripening and informed consumer choice. Moreover, integrating chemesthetic mechanisms with phenolic chemistry, olive ripening physiology, and sensory methodology allows for a more comprehensive interpretation of EVOO quality beyond commercial classifications. Future studies combining chemical profiling, dynamic sensory methods, and consumer-focused research will be essential to refine quality-assessment tools and promote a deeper appreciation of the sensory diversity and functional value of high-quality EVOOs.

## 1. Introduction

As established by the International Olive Council [1] and adopted by European Union regulations [2], virgin olive oils are intended for direct human consumption and designated as “*oils obtained from the fruit of the olive tree solely by mechanical or other physical means under conditions, particularly thermal conditions that do not lead to alterations in the oil, and which have not undergone any treatment other than washing, decantation, centrifugation and filtration*”. Regulation (EU) 2022/2104, as supplemented by Commission Regulation [2], defines three main commercial classes of olive oil, based on physical, chemical, and sensory features: extra virgin (EVOO), virgin (VOO), and lampante olive oil (LOO).

The proper extraction of EVOO from healthy and fresh olives makes it possible to obtain a product characterized by a unique fruity aroma and taste, and the absence of defects such as fusty, winey–vinegary, musty–humid, metallic, and rancid notes [3,4].

Esters, alcohols, ketones, and aldehydes (C5 and C6 compounds), synthesized through the lipoxygenase metabolic pathway, are responsible for EVOO’s fragrant and delicate flavor [5,6,7].

In addition, bitterness and chemesthetic sensations are typical sensory features of EVOO associated with the composition and concentration of phenolic secoiridoids and other bioactive compounds, belonging to the group of chemosensory active compounds (CACs) which contribute to EVOO’s sensory identity as well as to its well-recognized nutraceutical value [8,9]. The term ‘chemesthesis’ combines ‘chemical’ and ‘somesthesis’, and it refers to the chemical sensitivity of the skin and mucous membranes, perceived as pungency, irritation, warmth, cooling, or heat born by chemical molecules activating somatosensory neurons by specific chemical compounds [10].

Changes in sensory, nutraceutical, and chemical characteristics of EVOOs can be related to the raw material, affected by the technological process, or occur during storage mainly due to oxidative processes induced by light and oxygen exposure [11,12,13,14,15,16].

Among these factors, olive maturity is pivotal, since it profoundly influences the phenolic- and volatile profiles and, consequently, the sensory attributes of the final product.

This review aims to address chemesthetic perception in EVOO, highlighting the sensory mechanisms of trigeminal stimulation and the role of olive-fruit ripening as a key modulating factor through phenolic composition.

## 2. Main Sections

### 2.1. Chemesthetic Perception: Concepts and Mechanisms

Flavor perception is traditionally defined as the integrated output of the chemical senses of taste and smell. However, an indispensable component is trigeminal perception, which is responsible for both physical responses (e.g., temperature and texture) and chemically induced responses [17].

From a neurophysiological standpoint, taste and olfaction primarily rely on metabotropic G protein-coupled receptors (GPCRs), whereas trigeminal sensations are mediated by ionotropic transient receptor potential (TRP) channels [10,18,19].

TRP channels are expressed in nerve fibers and keratinocytes in the oronasal cavity, and some are also in taste buds. TRP channels play an integral role in transducing chemical stimuli into sensations such as irritation, warmth, coolness, and pungency, giving rise to chemesthetic sensations [10,19]. Since it is a chemical sense, initiated by molecular interaction, it is associated with the senses of taste and smell. In this context, some researchers are also investigating a potential implication of TRP channels in taste transduction [20,21,22].

Many chemical species present in food activate TRP channels expressed at the endings of trigeminal nerve fibers, leading to membrane depolarization and firing of action potentials in patterns distinct from those of taste- and olfactory pathways [10].

**Figure 1:**
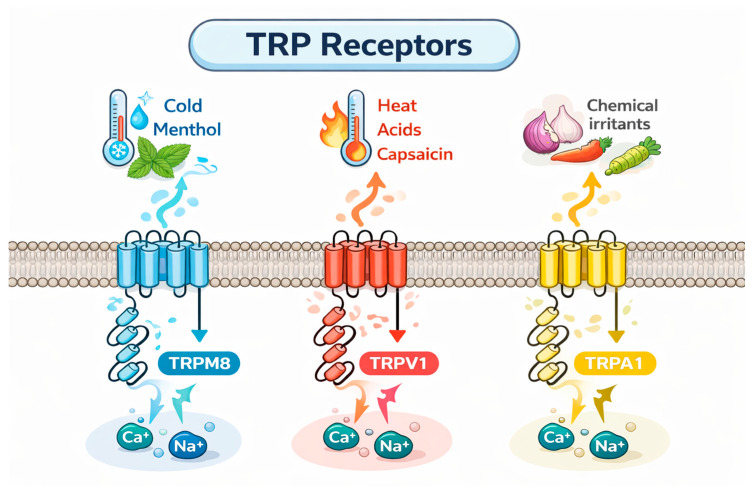
Simplified representation of TRP channels mainly involved in sensory chemesthesis. Activation of these non-selective cation channels induces Ca^2+^ and Na^+^ influx, leading to neuronal depolarization and the chemesthetic perceptions.

Regarding food perception, the most relevant TRP channels are TRPV1, TRPA1, and TRPM8 which are widely expressed on the trigeminal nerve fibers innervating the entire oral cavity but are probably expressed on all the sensory nerve fibers of the trigeminal nerve [19] (Figure 1).

TRPV1 is a non-selective cation channel activated by many physical and chemical stimuli such as noxious heat (T > 43 °C), low extracellular pH, divalent cations such as Mg^2+^ and Ba^2+^ as well as animal toxins, and chemical irritant compounds present in food like as capsaicinoids, piperine, and sanshool compounds [23]. Upon activation, TRPV1 allows sodium- and calcium ions influx, depolarizing nociceptive neurons, leading to action potential firing and eliciting the characteristic sensation [10,23,24,25,26].

TRPM8 is a polymodal ion channel, responsible for mediating the detection of cold thermal stimuli by primary afferent sensory neurons. It is activated by temperatures ranging from 8 °C to 26 °C and food compounds such as menthol, eugenol, and synthetic compounds such as icilin, as well as voltage changes and alteration in extracellular osmolarity [27,28].

Among the three TRP channels reported to be implicated in chemesthetic sensations, the least understood is TRPA1, which is activated by a broad range of stimuli, including electrophilic chemicals, oxygen, temperature, and mechanical force. TRPA1 transduces nociceptive signals associated with tissue damage and inflammation; it is implicated in itch pathways, in noxious cold and heat transduction, and activation induced by pungent compounds found in mustard oil and wasabi (e.g., allyl isothiocyanate), cinnamon (e.g., cinnamaldehyde), and, notably, oleocanthal from extra virgin olive oil [29,30].

Finally, TRPA1 and TRPV1 are known to be co-expressed in subsets of sensory neurons, a phenomenon that helps to explain the somewhat contradictory sensation of “burning cold” evoked at very low temperatures [31].

Among food-derived stimuli, EVOO is a unique natural source of TRPA1 and TRPV1 activators, making chemesthesis a central component of its sensory identity.

Although TRP channels represent the primary molecular substrates of chemesthetic perception, growing evidence [32,33,34,35,36,37] indicates that they do not fully account for the complexity of pungent sensations, suggesting the involvement of additional molecular targets.

In this context, Dawid et al. [38] demonstrated that piperine can activate a subset of trigeminal neurons even in the presence of TRP channel antagonists, indicating the involvement of alternative signaling pathways. Among the most plausible complementary targets are two-pore domain potassium (KCNK) channels, which have emerged as modulators of neuronal excitability in trigeminal neurons [32,33]. Notably, several KCNK channels have been identified in murine models as molecular targets of hydroxy-α-sanshool, a tingling-active compound, supporting their potential role in shaping chemesthetic sensations beyond canonical TRP-mediated mechanisms [39]. In line with this broader mechanistic perspective, a sensomic-based approach has demonstrated that complex pungent and tingling sensory impressions cannot be attributed to a single molecular target. In the case of black pepper, Dawid et al. [38] identified multiple CACs responsible for pungent and tingling sensations and showed that several of these compounds markedly modulate two-pore domain potassium channels (KCNK).

According to these findings, it seems that KCNK channels may act as complementary targets to TRP channels in defining chemesthetic perception, contributing to the complexity of oral responses induced by pungent foods [33,38,40]. In particular, KCNK channels generate background potassium “leak” currents that regulate neuronal excitability by stabilizing the resting membrane potential and modulating action potential firing in sensory neurons [41,42,43]. Modulation in KCNK channel activity can therefore influence the response threshold of sensory neurons to CACs, contributing to the perception of pungency and tingling sensations.

**Figure 2:**
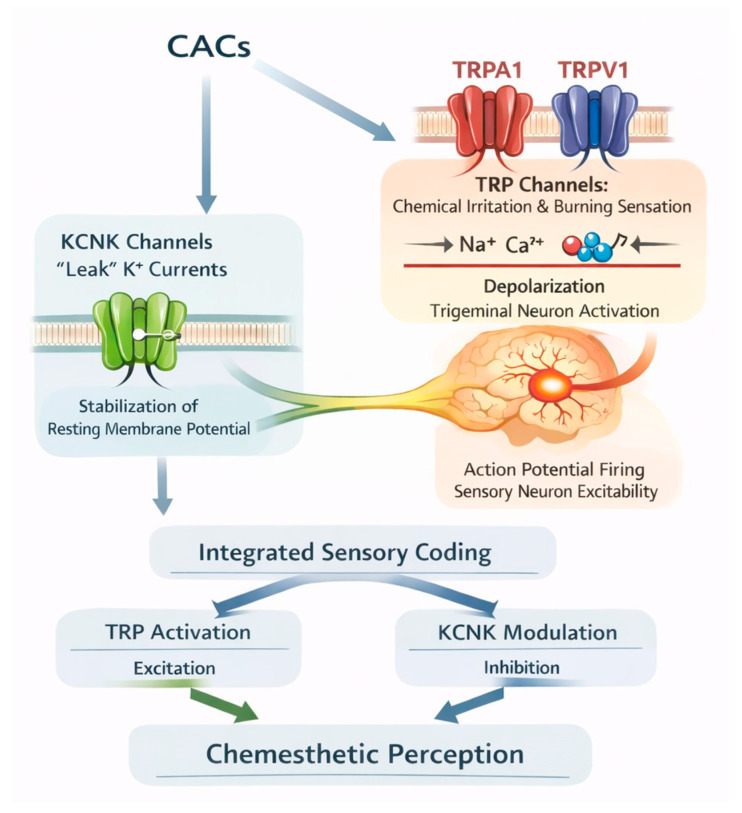
Simplified representation of sensory neuron responses induced by CACs highlighting the combined contribution of TRP channel activation and KCNK channel modulation to neuronal excitability.

Although TRP and KCNK channels operate through distinct mechanisms, with TRP channels mediating depolarizing cation influx and KCNK channels providing hyperpolarizing leak conductance, their combined action contributes to the overall excitability of trigeminal afferents (Figure 2). In this context, TRP channel activation enhances depolarizing drive, while concurrent KCNK channel inhibition reduces membrane stabilization, thereby facilitating action potential firing.

It should, however, be emphasized that current evidence supporting this multi-channel framework mainly derives from studies on other pungent foods [33,42,44]. Accordingly, the proposed scheme should be regarded as a theoretical and integrative model rather than a fully established mechanism specific to EVOO. Nevertheless, this perspective provides a useful conceptual basis for interpreting the complexity of EVOO chemesthetic perception.

### 2.2. Bioactive Compounds Underlying Chemesthetic Sensations in EVOO

The flavor of EVOO is an integrated perception of odor (both orthonasal and retronasal), taste, and chemesthetic sensations (e.g., tingling or warming sensation, pungency and astringency) elicited by chemosensory active compounds. The sensory profile of EVOO is markedly different from that of other edible lipids, thanks to its specific mechanical extraction process which avoids refining [45].

Regarding its composition, EVOO is made up of a saponifiable fraction for about 98–99% of the oil, while the remaining 1–2% is the unsaponifiable fraction [46]. Although quantitatively limited, the unsaponifiable fraction contains a wide range of both polar and non-polar compounds [47,48] (e.g., esters, phenolic compounds, pigments, volatile molecules) which play a pivotal role in defining the sensory characteristics of EVOO [49,50,51] (Table 1).

One of the most distinctive features of EVOO is its chemesthetic profile, characterized by pungency, astringency, and a tingling or warming sensation of variable intensity.

Pungency refers to a sharp, biting, and often persistent sensation evoked by the stimulation of trigeminal and other free nerve endings in the nasal or oral epithelium [60]. This phenomenon is characterized by a burning, peppery-like sensation that manifests when oil reaches the throat following ingestion. However, it has been observed that a burning sensation may be experienced in the mouth upon ingestion of certain olive oils [54].

Astringency is defined as “*the complex of sensations due to shrinking, drawing or puckering of the epithelium as a result of exposure to substances such as alums or tannins*” [59,61] and is commonly perceived as dryness, roughness, and lapping throughout the oral cavity [53,62].

Tingling, a less frequently reported sensation in EVOO, is described as a prickling or buzzing sensation on the tongue and is often perceived as a mild numbing or warming sensation [55]. While alkylamides of Szechuan pepper are well-known elicitors of tingling [23], compounds derived from oleuropein aglycone have been reported to induce tingling or numbing in EVOO through modulation of mechanosensitive neurons, partly via inhibition of two-pore domain potassium (KCNK) channels [55,63].

Chemesthetic sensations in EVOO arise from CACs that largely coincide with bioactive phenolic compounds associated with important health benefits [45].

Within the EVOO phenolic fraction, simple phenols represent only a small portion, whereas the main contributors to chemesthetic perception are secoiridoids, including oleocanthal, oleacin, and derivatives of oleuropein and ligstroside which contain tyrosol and hydroxytyrosol moieties in their molecular structure [53,55,64].

The concentration of these compounds in EVOO is strongly influenced by genetic factors, fruit ripening stage, and technological conditions during oil extraction [14,15,65].

Some phenolic compounds contributing to bitterness may also elicit astringency, through interactions with salivary proteins and pungency through stimulation of trigeminal nociceptive fibers, which transmit thermal, tactile, and pain-related sensations [64,66,67,68,69]. Moreover, phenolics can act synergistically with other components of both the saponifiable and non-saponifiable fractions. Free fatty acids such as linolenic- and linoleic acids have been reported to enhance the perception of burning, pungency, and bitterness [6].

**Figure 3:**
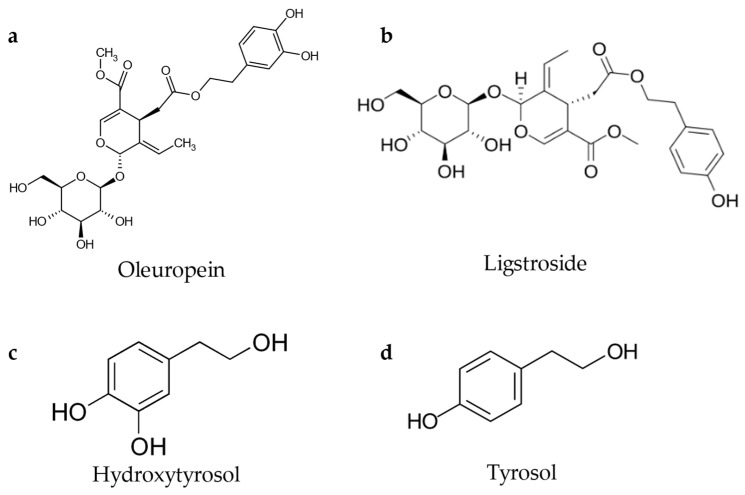
Chemical structure of oleuropein (**a**), ligstroside (**b**), hydroxytyrosol (**c**), and tyrosol (**d**).

As previously discussed, the most significant portion of the phenolic fraction is represented by aglycons derived from the secoiridoid glucosides oleuropein (Figure 3a) and ligstroside [52,60,64,70](Figure 3b), whose glycosylated form originated from the condensation of their respective precursors, hydroxytyrosol (Figure 3c), or tyrosol (Figure 3d), with intermediates of the secoiridoid biosynthetic pathways.

**Figure 4:**
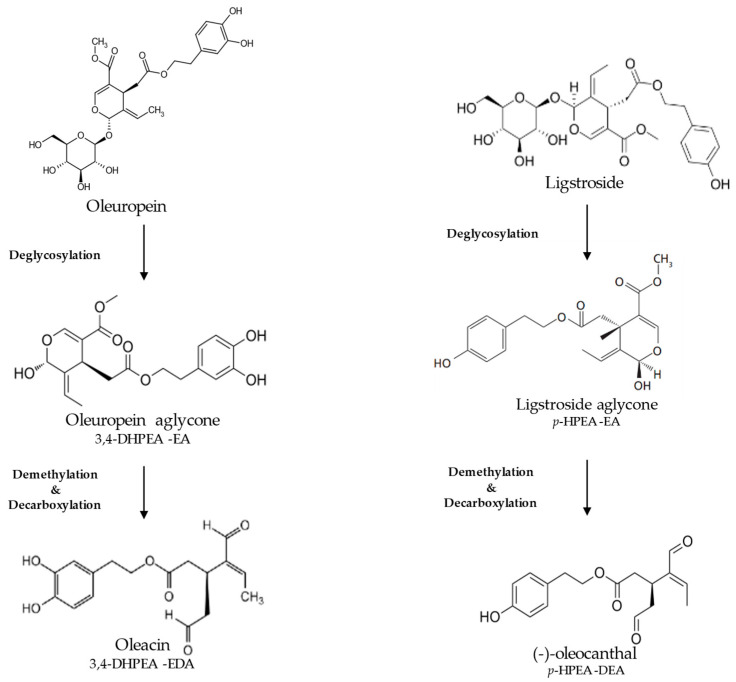
Genesis of oleacin and oleocanthal starting from oleuropein and ligstroside, respectively.

During EVOO extraction, crushing disrupts cellular compartments and releases endogenous enzymes such as *β*-glucosidase, which catalyzes deglycosylation followed by demethylation and decarboxylation of oleuropein (Ole) and ligstroside, leading to the formation of their dialdehydic forms oleacin (OL) and oleocanthal (OC), considered the main drivers of the chemesthetic sensations of pungency and numbing [9,52,54,71,72,73] (Figure 4).

Oleacin (OL) has been associated with mild burning/tingling sensation, mainly perceived on the tongue, and some of its derivatives have also been implicated in astringency [52,55,60,70].

Oleocanthal (OC), by contrast, elicits a strong, spatially localized burning sensation in the oropharynx, often followed by coughing and throat clearing; this sensation typically fades gradually after swallowing. OC acts as an agonist of the TRPA1 channel, specifically the human TRPA1 isoform [66,74,75], although the reason underlying its distinctive perceptual signature remains incompletely understood [44,55,76,77,78]. In contrast to what has been reported for many TRPA1 agonists, which typically activate the channel through covalent modification of cysteine residues [79,80], OC does not appear to rely on this canonical activation mechanism [74]. It has been shown that TRPA1 can remain responsive to OC even when certain cysteine residues are rendered non-reactive, suggesting the involvement of an alternative mode of activation [74]. Overall, astringency emerges as a complex, multifaceted sensation influenced by multiple variables and mediated through both mechanical- and chemical pathways [61]. Although not included among the official IOC [1] positive attributes, it is consistently reported in EVOO and is mainly associated with interaction between phenolic compounds and salivary proline-rich proteins. Recent evidence suggests that mechanosensitive pathways may play a predominant role in this perception [81]. Its intensity generally increases with phenolic concentration and contributes significantly to the overall mouthfeel of high-phenolic EVOOs. Compounds of EVOO reported to be responsible for astringency are tyrosol, OC and OL, and isomers of ligstroside and oleuropein aglycone [55].

### 2.3. Influence of Olive Maturity on Chemesthetic Compounds

**Figure 5:**
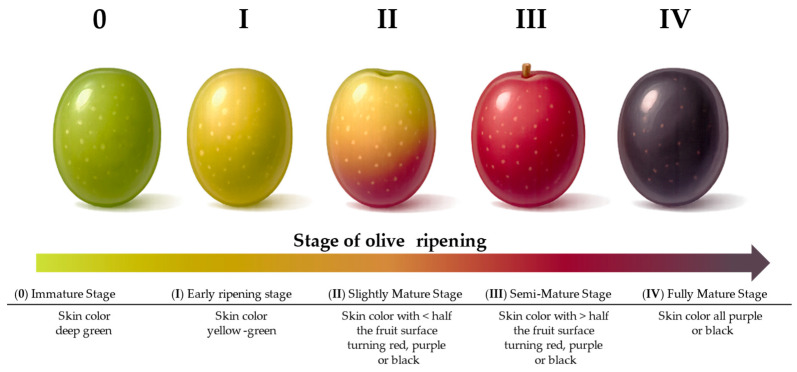
Stages of olive-fruit ripening. Schematic representation of the five ripening stages of the olive fruit as described by Uceda and Frias [82]: (0) Immature Stage (I) Early Ripening Stage (II) Slightly Mature Stage (III) Semi-Mature Stage (IV) Fully Mature Stage.

Olive-fruit ripening results from a combination of tightly regulated physiological and biochemical pathways that are strongly conditioned by pedoclimatic and agronomic conditions, as well as by cultivar-dependent genetic factors [65]. The ripening stages of olive fruit are associated with progressive changes in fruit morphology, weight, color, metabolic activity, and in the composition of sensory-related biochemical compounds. The most widely adopted method for evaluating olive fruit maturity is the maturity index (MI) proposed by Uceda and Frías [82], which is based on the visual classification of olives according to skin color and the degree of color penetration into the flesh [61,83]. In Figure 5, the first five ripening classes defined considering skin color are illustrated.

Among these metabolic changes, the evolution of phenolic compounds is particularly relevant, as their biosynthesis, transformation, and degradation during ripening strongly depend on both enzymatic activity and genetic background, ultimately influencing the chemesthetic properties of EVOO [65].

A general decrease in bitterness and pungency during ripening has long been observed [60,84], reflecting the reduction in total phenols and *o*-diphenols levels, as well as in the secoiridoids [65].

Polyphenol biosynthesis is most active in the early stages of olive development and ripening, reaching a maximum level around the half-pigmentation point [53,65,84]. Then, the biosynthetic capacity of the olive declines [85].

However, this behavior is not uniform and varies according to cultivar specific genetic traits, climatic, and seasonal conditions [67,85,86]. In some cultivars, an increase in total phenol content in advanced ripening stages occurs, mainly linked to the accumulation of anthocyanins, which are used as indicators of fruit maturation. Biosynthesis of anthocyanins is typically accompanied by a decrease in bitter secoiridoids, driven by increased enzymatic activities during ripening, and producing phenols of lower molecular weight responsible for chemesthetic sensation [84,87].

*β*-glucosidase plays a central role in the hydrolysis of phenolic glucosides, including Ole, ligstroside, demethyl-ligstroside, and demethyl-oleuropein, generating aglycones that are precursors of CACs such as OL and OC [67,85,86,87,88].

Oxidative enzymes, such as polyphenol oxidase (PPO) and peroxidases, may also participate in phenolic degradation; however, their activity does not consistently correlate with secoiridoid levels, suggesting that oleuropein decline is driven primarily by glycosidase activity by metabolic regulation that varies across cultivars due to their distinct genetic backgrounds rather than by PPO-mediated oxidation [89]. During ripening, additional chemical and enzymatic reactions yield lower-molecular-weight phenolics, further modifying the chemesthetic potential of the fruit.

**Figure 6:**
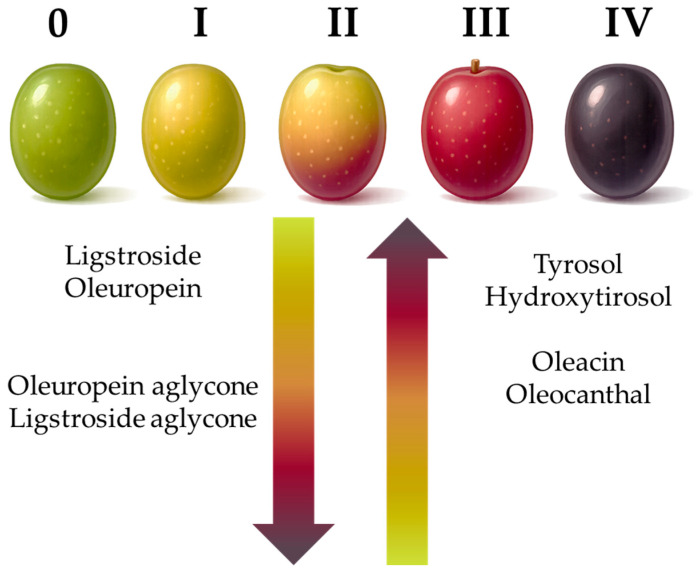
General behavior of principal CACs and their precursors during olive ripening.

While simple phenol content tends to remain quite stable throughout the olive ripening [8,90,91,92], the fate of secoiridoids is more evident (Figure 6). In most cultivars, oleuropein content progressively decreases during ripening, due to glycosidase activity, and is replaced by demethyl-oleuropein and hydroxytyrosol [67,86,88].

Ligstroside is mainly found in early green olive fruits, with its concentration progressively decreasing as the fruit develops, until it is present only in trace amounts in fully black olives [93]. Ole and its aglycone accumulate from green to black olives rising by 30% and 40%; the dimethyl oleuropein content grows by up to 75%; during olive ripening the content of free hydroxytyrosol increases by 20%, while the tyrosol level roughly doubles in black olives [93]. The concentration of hydroxytyrosol elenolate and oleuropein aglycone are reported to decrease, reaching nearly half of their initial content in black olives [93]. As the Ole content decreases, the concentration of its glucosylated derivatives begins to appear and they subsequently accumulate, reaching their highest concentrations at the black maturation stage, when oleacin becomes one of the predominant phenolic constituents [94].

To summarize, although some varieties, such as the Gemlik, show non-linear trends, the general pattern observed across several studies shows a marked reduction in Ole content, accompanied by a parallel increase in hydroxytyrosol- and antioxidant capacity in late ripening [67].

Genotype-dependent regulatory differences in secoiridoid biosynthesis and catabolism explain these variable accumulation patterns: high-phenolic cultivars maintain higher expression of biosynthetic genes than low-phenolic ones, and most transcripts involved in secoiridoid biosynthesis decrease as fruit development progresses [95].

These biochemical- and cultivar-dependent regulatory processes ultimately modulate both the quantity and the structural diversity of chemesthetic-active secoiridoids released into EVOO during extraction [50,52,87,96]. Oils obtained from early-harvest fruits, which retain a larger pool of intact secoiridoid precursors, generally exhibit higher levels of OC and OL and therefore display more intense pungency and the characteristic throat-sting associated with TRPA1 activation.

In contrast, as ripening progresses, the progressive hydrolysis, oxidation, and catabolism of secoiridoids reduce the formation of these aglycones, leading to oils with a milder trigeminal impact and a less complex chemesthetic profile. Such sensory attenuation is particularly evident in cultivars with inherently lower phenolic potential or faster phenolic degradation dynamics [50,52].

In Cornicabra, for example, Ole content in olive fruits decreases strongly throughout the ripening process, dropping from values above 11,000 mg/kg at early maturity (ripening index RI = 1) to around 6300 mg/kg at full ripeness (RI4), resulting in a final decrease in total phenol content of 17% [97]. Regarding the chemesthetic potential, oils obtained from Cornicabra olives exhibit high levels of OC and OL, which remain above 300 and 700 mg/kg, respectively, even at advanced ripening stages. Regarding HT, which is reported as the main contributor to astringency, Cornicabra olive fruits showed an overall decrease of approximately 13% during ripening, (271 to 236 mg/kg), resulting in a low concentration in the corresponding oils (around 3–2 mg/kg).

The same authors [97] reported a similar pattern for Picual olives, where Ole content decreases by 26% during ripening (from ≈8000 to ≈6000 mg/kg) despite a transient increase at the intermediate stage (RI2.5 ≈ 8300 mg/kg), while the resulting oils retain a high total phenol content (≈900 mg/kg) and relevant OL (from 230 to ≈200 mg/kg) and OC (350 to ≈330 mg/kg) concentrations. In contrast to Cornicabra, Picual olives exhibit an increment of HT during ripening (≈330 to ≈380 mg/kg), reflected in the oils’ HT concentration (≈1.8 to 2.2 mg/kg).

In contrast, naturally low-phenolic cultivars such as Arbequina show a limited chemesthetic potential already at early maturity. In Arbequina fruits, Ole content is relatively low even at early ripening stages (RI1 ≈ 2200 mg/kg) and undergoes a sharp decrease during maturation, reaching values below 100 mg/kg at full ripeness. This decrease is accompanied by a progressive increase in HT (from ≈200 at RI1 to ≈350 mg/kg at RI4), which is only marginally reflected in the corresponding oils, where HT is at very low levels (≈2–3 mg/kg). Consistently, Arbequina oils has low concentrations of OL and OC, decreasing from around 200 and 140 mg/kg, respectively, at early harvest to around 125 and 95 mg/kg at full ripening stages [97].

Table 2 summarizes the main cultivar-dependent trends in phenolic content and secoiridoid dynamics reported across olive ripening stages. The trends reported are qualitative and provide a comparative synthesis of consistent patterns observed across the literature, rather than quantitative values, due to the heterogeneity of cultivars and methodologies among studies. In this context, chemesthetic potential refers to the relative propensity of the resulting EVOO to elicit chemesthetic sensations, derived from phenolic composition, secoiridoid profile, and their evolution during ripening. It represents a qualitative and comparative indicator rather than a direct or quantitative sensory measurement, as actual perception is further modulated by processing conditions [98], matrix effects [74], and individual sensitivity [99]. Although cultivar-dependent trends in phenolic composition can be identified, establishing direct- and quantitative relationships between olive fruit phenolics and EVOO sensory profiles remains challenging due to the biological and technological factors, affecting phenolic transformation, as well as the complexes mechanisms underlying sensory and chemesthetic perception.

The reported patterns highlight the combined influence of genetic background, environmental conditions, and ripening physiology on the chemesthetic properties of EVOO. The wide variability in pungency observed among EVOOs reflects not only biological- and environmental factors but also agronomic- and production-related choices. Differences in pungency levels among EVOOs produced in different regions or by different producers arise from the interplay of cultivar-dependent genetic traits, pedoclimatic conditions, and technological variables [50,100]. Phenolic biosynthesis and secoiridoid accumulation, which shape the potential chemesthetic profile of the oils, are strongly influenced by genetic background and environmental conditions [101], while harvest timing and processing choices could be deliberately adjusted to achieve specific sensory styles, in response to regional traditions, market positioning, and targeted consumer segments. Accordingly, the wide range in pungency observed across EVOOs can be interpreted as the results of intrinsic cultivar-related factors and intentional production strategies aimed at meeting regional traditions, market positioning, and targeted consumer segments.

### 2.4. Chemesthesis, Phenolic Bioactivity, and Nutraceutical Relevance of EVOO

The great interest in EVOO phenolics is due to their association with a wide range of biological activities. The unique overlap between sensory chemesthesis and nutraceutical potential distinguishes EVOO from other edible fats: the same compounds responsible for pungency and tingling, mainly mediated by TRPA1 and TRPV1 activation, also contribute substantially to the health-promoting properties attributed to EVOO [102].

Among these compounds, secoiridoid derivatives that elicit chemesthetic sensations, most notably oleocanthal and oleacin, represent some of the most biologically active phenolic constituents of the oil [8,72,90,97,102]. Several studies have demonstrated beneficial effects of these purified secoiridoids from EVOO in the context of metabolic syndrome, cardiovascular disorders, inflammatory bowel diseases, skin disorders, and infectious diseases, providing a mechanistic basis for the health benefits associated with EVOO consumption [72,76,97,102,103].

Ole is one of the most extensively studied secoiridoids and has been reported to exert hypolipidemic effects, together with OL, as well as to modulate molecular mechanisms involved in metabolic, cardiovascular, and neurological diseases, and cancer [72,104]. Ole also exhibits antimicrobial activity against several bacterial strains responsible for intestinal- and respiratory-tract infections in humans, an effect similarly reported for hydroxytyrosol [103]. In addition, oleuropein has been proposed as a potential therapeutic agent for inflammatory bowel diseases and skin disorders [72].

OL likewise displays a broad spectrum of biological activities including hypolipidemic, antihypertensive, and vasodilatory effects [72] and shares with OC notable anti-inflammatory, antioxidant, and immunomodulatory properties. The anti-inflammatory activity of both OL and OC is linked to reversible inhibition of cyclooxygenase (COX)-1 and COX-2 in a manner analogous to ibuprofen, suggesting their potential relevance in inflammatory- and reactive oxygen species (ROS)-related diseases [57,72,76].

OC has also been widely studied for its activity against neurological disorders and cancer and has attracted considerable interest due to its strong anti-inflammatory properties and its ability to modulate immune responses, reinforcing its potential role in the prevention and management of chronic inflammatory conditions [57,72,102,105].

Together, these secoiridoids form the biochemical foundation of the EFSA-authorized health claim concerning the protective role of olive phenolics against oxidative stress [106]. It is interesting that chemesthetic intensity often is in line with the nutraceutical value. Oils extracted from early-harvest olives, with higher concentrations of secoiridoid precursors, generally exhibit pronounced pungency/throat-sting. Conversely, oils obtained from very ripe olives, characterized by the progressive degradation of secoiridoids and increased formation of hydroxytyrosol and tyrosol, exhibit milder chemesthetic sensations and lower concentrations of secoiridoids. Thus, chemesthetic perception may act as a sensory indicator of phenolic richness and biological efficacy, as reported by some authors [50,52,54].

Furthermore, as described in Section 2.3, high-phenolic cultivars (e.g., Picual, Cornicabra, and Morisca) maintain high secoiridoid biosynthesis and stronger chemesthetic expression [89,93,95], while naturally low-phenolic cultivars (e.g., Arbequina and Chemlali) generate oils with weaker trigeminal impact and also lower nutraceutical activity [88,97].

### 2.5. Sensory Assessment of Chemesthetic Perception in EVOO

#### 2.5.1. Methodological Approaches for Chemesthetic Evaluation

Sensory analysis is crucial in assessing the quality of EVOO, by evaluating attributes such as color, aroma, taste and trigeminal sensation, which depend on both intrinsic factors and production practices.

Virgin olive oils are officially classified through the “IOC Panel Test”, developed and continuously refined by the International Olive Council (IOC), which is based on trained assessors, operating under controlled conditions to evaluate the intensity of sensory defects and of the main positive attributes (i.e., fruitiness, bitterness and pungency) using standardized descriptors and continuous intensity scales [107,108].

This approach is indispensable for regulatory classification; anyway, it does not fully describe the complexity of EVOO sensory profile [109].

To overcome these limitations, descriptive sensory analysis has been widely adopted, allowing trained assessors to measure the intensity of a broader range of sensory attributes, such as olfactory, retronasal, gustatory, chemesthetic (e.g., pungency and astringency), and tactile sensations [110].

Evaluation forms are tailored to the objectives of the study, employing continuous scales, while results are statistically analyzed to assess sample differences and panel reproducibility [107].

Bitterness, pungency, and astringency are characterized by delayed onset, long persistence, and strong carry-over effects; therefore, the number of samples that can be reliably evaluated in a single session is generally reduced and an adequate rest period between samples is necessary [111,112]. Moreover, these sensations do not emerge simultaneously, making dynamic approaches especially relevant for chemesthetic profiling (e.g., time–intensity methods); firstly, bitterness reaches its maximum intensity (≈16–20 s), then pungency (≈26–29 s), while astringency may persist for more than 60 s [109,110,112,113].

Chemesthetic attributes have a slower sensory recovery and may induce phenomena of trigeminal sensitization and desensitization, which can increase data variability if evaluation protocols are not rigorously standardized; therefore, they need specific methodological challenges compared to aroma and taste evaluation [114,115]. The timing of evaluation is particularly critical, since chemesthetic sensations may differ markedly when assessed at first contact, during oral processing, or as aftertaste, and their temporal evolution can vary substantially among samples [115,116].

Therefore, dynamic sensory approaches have been increasingly applied to EVOO to better capture the temporal evolution of bitterness and pungency and to improve methodological standardization, particularly in phenols-rich oils [15,69].

Recent studies have evaluated and refined temporal evaluation techniques such as Temporal Dominance of Sensations (TDS), Temporal Check-All-That-Apply (TCATA), and their combinations with liking measures, demonstrating enhanced discriminative power and richer temporal profiling of multiple sensory attributes in consumer panels [117,118].

In this context, dynamic sensory methods, such as time–intensity (TI) analysis, seem to be effective for characterizing chemesthetic perception [60]. TI methods allow the temporal evolution of attributes to be captured, obtaining parameters such as maximum intensity, time to maximum, duration, and total perceptual impact [114,115,116]. This approach is particularly interesting for EVOO, characterized by compounds such as oleocanthal, which is defined by intensity and by delayed onset, localization in the oropharynx, and gradual decrease over time. Differences in temporal evolution may help to discriminate oils obtained from different cultivars or harvested at different ripening stages.

#### 2.5.2. Consumer Perception and Acceptance of Chemesthetic Attributes

Chemesthetic attributes of EVOO (e.g., pungency and astringency) are widely recognized by experts and trained EVOO tasters as positive sensory markers of freshness, phenolic richness and quality. However, most consumers are unfamiliar with this sensory–qualitative relationship and often interpret intense bitterness or throat-sting as defects rather than as indicators of bioactive compounds with nutritional relevance [60].

Excessive chemesthetic intensity, typically associated with early harvesting or very high phenolic levels, may therefore reduce acceptability, particularly among individuals with heightened sensitivity to trigeminal stimuli [52]. In this context, consumer pleasantness and acceptance of EVOO are strongly influenced by the phenolic concentration, which determines the intensity of bitterness and pungency: oils rich in secoiridoids, such as OC and OL, often display remarkable sensory complexity and health benefits, but may be rejected by untrained consumers in favor of milder profiles characterized by green fruity notes and light-to-medium bitterness and pungency. On the contrary, consumers which generally consume lower-quality oils may confuse negative attributes such as rancidity or mustiness with normal sensory characteristics [54,119]. Consumer acceptance of bitter- and pungent attributes in extra virgin olive oil is reported to be strongly influenced by familiarity and cultural context, with consumers from olive-oil-producing regions showing a significantly higher appreciation of these sensory cues compared to consumers from low-familiarity areas, where the same attributes are often perceived negatively [120].

Many studies have reported that expert sensory evaluation does not match consumer preferences. This gap is mainly due to consumer familiarity with sensory attributes, individual differences in sensitivity, and low exposure to EVOOs with high phenolic content [54,111,119]. Furthermore, acceptance of chemesthetic sensations can change over time: repeated exposure and a better understanding of the health role of phenolics compounds may lead consumers to prefer oils with stronger sensory profiles [54,112]. In this regard, information can be a crucial instrument. A study performed by Cardillo in 2025 [121] showed that when the nutritional benefits of high phenolic EVOOs are shown to consumers, consumers’ preferences change rapidly.

This fast preference change has been linked to the tendency of people to accept a less-pleasant taste when the aim is to make a healthy choice [121,122].

Recent studies also demonstrate that clearer and more transparent label information, such as harvest year, cultivar, and extraction method, can improve consumers’ ability to align sensory expectations with actual product quality, thereby helping to reduce the gap between expert sensory evaluation and consumer acceptance [123,124].

These aspects highlight the importance of consumer education, helping people better understand EVOO sensory quality and its relationship with phenolic composition and antioxidant properties.

While sensory analysis is traditionally used for olive oil classification, product authentication, and early detection of quality defects, it also holds significant potential as an educational tool to train consumers in recognizing quality attributes. In this context, nutritional [125] and sensory education could play a pivotal role in bridging the gap between sensory perception and olive oil quality. One approach to achieve this goal in adult consumers could be the introduction of specific health claims explicitly linked with specific sensory cues, such as pungency and bitterness. As reported by Fakhreddine and Sanchez [126], health claims can change consumer purchasing intentions in the EVOO market. Another important step would be the introduction of sensory- and nutrition education in basic school with the aim of raising a new generation of more conscious and informed consumers.

A complementary tool to modulate chemesthetic intensity while preserving phenolic richness could be represented by product formulation and blending approaches. Adjusting cultivar selection, harvest timing, blending ratios, and technological approaches [98,127,128,129,130,131], can be properly modulated to design EVOOs with differentiated chemesthetic profiles that better meet the expectations of specific consumer segments. Although analytical techniques have improved substantially in correlating chemical composition with sensory attributes, human sensory evaluation remains irreplaceable, as the multidimensional flavor of EVOO arises from the interaction of numerous volatile and non-volatile compounds whose combined perceptual effects cannot be fully captured by instrumental methods alone.

## 3. Conclusions

Chemesthetic perception is a distinctive component of the sensory identity of EVOO, linked primarily to phenolic secoiridoids which also are responsible for its health-promoting properties. As previously discussed, pungency, tingling, and astringency sensations are not only sensory descriptors, but are related to the presence, structure, and bioactivity of phenolic secoiridoids.

Olive ripening is a pivotal factor in modulating both the qualitative- and quantitative profile of CACs. Early-harvest olives generally yield oils richer in secoiridoid derivatives and therefore are characterized by stronger chemesthetic sensations and higher nutraceutical value, whereas olives harvested at advanced ripening stages yield oils with milder sensory profiles due to the progressive phenolic degradation.

These effects are modulated also by cultivar-dependent genetic regulation, which justifies the wide sensory diversity observed among EVOOs even within the same commercial category.

From a sensory-science point of view, evaluating chemesthetic attributes involves specific methodological difficulties. These sensations change over time, can last for a long period, and often cause carry-over effects between samples. Although the IOC panel test is essential for regulatory classification, it is not well-suited to describe the dynamic- and spatial aspects of chemesthetic perception. For this reason, descriptive sensory analysis and dynamic approaches, such as time–intensity evaluation, are useful tools to better capture the complexity of EVOO sensory quality, especially for sensations that appear later and are mainly perceived in the oropharyngeal area.

The frequent mismatch between expert sensory evaluation and consumer acceptance also highlights the need to improve how chemesthetic attributes are explained and communicated. Bitterness and pungency are often perceived negatively by untrained consumers, but several studies indicate that acceptance can change with repeated exposure, learning, and a better understanding of the health role of phenolic compounds. In this sense, chemesthetic perception can be considered not only a quality-related sensory feature, but also an indicator of phenolic richness and biological potential, helping to link sensory science with informed consumer choice.

Improving consumer awareness of sensory perception and its relationship with health-related claims may therefore enable more informed food choices.

Consumer education may play an important role in improving the acceptance of chemesthetic attributes in extra virgin olive oil, as familiarity and repeated exposure are known to influence sensory appreciation and preference formation. Educational approaches based on sensory training and guided tasting experiences could help consumers reinterpret bitterness and pungency not as defects, but as positive quality cues associated with phenolic composition and potential health value. In this perspective, the inclusion of basic food- and sensory education within school curricula may represent a promising long-term strategy to foster early familiarity with bitter and pungent flavors and to reduce food neophobia. More broadly, multi-level educational initiatives involving schools, gastronomy professionals, and digital communication tools could contribute to narrowing the gap between expert sensory evaluation and consumer perception of EVOO quality.

Overall, combining chemesthetic mechanisms with phenolic chemistry, olive ripening physiology and sensory evaluation methods offers a more complete way to interpret EVOO quality beyond traditional classifications.

Future studies that integrate chemical analysis, dynamic sensory approaches, and consumer-focused research will be important to improve quality-assessment tools and to support a broader appreciation of the sensory diversity and functional value of high-quality extra virgin olive oils.

## Figures and Tables

**Table 1:** Qualitative overview of the main sensory attributes of EVOO and the chemical compounds most commonly associated with them.

Sensory Attribute	Perceptual Modality	Key Compounds	Notes	Ref.
Green, fruity, herbaceous aroma	Olfaction	C6 aldehydes (hexanal, E-2-hexenal), C6 alcohols (hexanol), C5 compounds, esters, ketones	Formed via lipoxygenase pathway during crushing; responsible for “green” and “fresh-cut grass” notes	[5,52,53,54]
Floral, ripe fruit aroma	Olfaction	Esters, terpenes, β-ionone, volatile lactones	Associated with ripeness-related metabolic changes	[5,52,53]
Bitterness	Taste (gustatory)	Oleuropein aglycone, ligstroside aglycone, simple phenols (hydroxytyrosol derivatives)	Correlates with phenolic concentration; positive attribute in high-quality EVOO	[55,56,57]
Pungency (“throat-sting”)	Chemesthesis	Oleacein (oleuropein-aglycone dialdehyde derivative), minor secoiridoids	Strong activation of TRPA1 channels; typical “throat burn”	[53,55,57]
Tingling/warming sensation	Chemesthesis	Oleacein (oleuropein-aglycone dialdehyde derivative), minor secoiridoids	Milder burning/numbing sensation; activation of KCNK two-pore potassium channels; partly TRPV1-mediated	[53,55,57]
Astringency	Chemesthesis/tactile	Simple phenols (tyrosol, hydroxytyrosol), phenolic acids, and certain secoiridoids	Interaction with salivary proteins, leading to dryness, roughness, puckering	[53,55,57]
Mouthcoating/viscosity	Tactile/somatosensory	Triacylglycerol composition; unsaponifiable fraction	Affects mouthfeel	[53]
Mold-humidity	Olfaction	pungent, mushroom (1–octen–3–one); mold (1–octen–3–ol); sweet, cinnamon (2–heptanone); earthy (2–heptanol);	Off-flavors caused by the presence of fungi and yeast in olives. Used in sensory panel classification; EVOO must be free of all sensory defects	[53,54,58]
Winery-vinegar	Olfaction	Alcohol (ethanol); sweet (ethyl acetate); woody, whiskey (3–methylbutanol); sour (acetic acid);	Off-flavors caused by aerobic fermentation in olives or olive paste. Used in sensory panel classification; EVOO must be free of all sensory defects	[53,58,59]
Fusty	Olfaction	fruity, green, pungent (butyl acetate); fruit (ethyl propanoate); sweet (ethyl butanoate); pungent (propanoic acid)	Off-flavors related to extensive anaerobic fermentation in olives or to oil in contact with the sediment. Used in sensory panel classification; EVOO must be free of all sensory defects	[53,54,58]
Muddy-sediment	Olfaction	rancid, cheese (butanoic acid); unpleasant (pentanoic acid)	Off-flavors related to extensive anaerobic fermentation in olives or to oil in contact with the sediment. Used in sensory panel classification; EVOO must be free of all sensory defects	[53,54,58]
Rancidity	Olfaction	oxidized, pungent (trans–2–heptenal); herbaceous (trans–2–octenal); fishy (trans–2–decanal); woody, bitter, (pentanal); fatty, green (hexanal); oily, woody (heptanal); sharp (octanal); waxy (nonanal); rancid, (hexanoic acid); rancid (heptanoic acid); herbaceous (6–methyl–5–hepten–2–one)	Off-flavors, related to oils strongly oxidated. Used in sensory panel classification; EVOO must be free of all sensory defects	[53,54,58]

**Table 2:** Qualitative cultivar-dependent trends in phenolic evolution, secoiridoid dynamics, and relative chemesthetic potential of EVOO across olive ripening stages.

Cultivars	Phenolic Trend	Secoiridoid Behavior	Chemesthetic Potential in EVOO	Key References
Ayvalık	Progressive decrease in total phenols	Ole decreases, HT increases	High at early ripening, decreasing to medium at later stages	[67]
Gemlik	Non-linear trend	Ole increases at early stages and decreases at late ripening	Variable	[67]
Chemlali	Progressive decrease	Ole decreases, HT increases	Medium at early stages, low at full ripening	[88]
Dhokar	Progressive decrease	Ole progressively decreases	Medium at early stages, low at full ripening	[88]
Arbequina	Low baseline phenolic content, decreasing during ripening	Increase in demethyloleuropein; verbascoside remains stable	Low at early stages, very low at full ripening	[97]
Cornicabra	Moderate decrease	High Ole levels at early stages, decreasing with ripening	High at early ripening, decreasing to medium	[97]
Picual	Moderate decrease	Strong correlation between fruit and oil secoiridoid content	High at early ripening, decreasing to medium	[97]
Picudo/Picolimón	Moderate decrease	Similar to Picual	Medium-to-high, depending on ripening stage	[89,97]
Morisca	Moderate decrease	Variable by season and climate	Medium, with cultivar- and season-dependent variation	[97]
Nocellara Etnea	Low baseline phenolic content, decreasing during ripening	OL content decreases during ripening	Low at early ripening, decreasing to very low	[95]
Dritta	Low baseline phenolic content, decreasing during ripening	Strong genetic control of phenolic pathway	Low	[95]
Tendellone	Low baseline phenolic content, decreasing during ripening	OL content decreases to near zero at full ripening	Medium at early stages, low at full maturity	[95]

## Data Availability

No new data were created or analyzed in this study. Data sharing is not applicable to this article.

## Abbreviations

The following abbreviations are used in this manuscript:

IOC	International Olive Council
EVOO	Extra Virgin Olive Oil
VOO	Virgin Olive Oil
LOO	Lampante Olive Oil
TRP	Transient Receptor Potential
CACs	Chemosensory Active Compounds
Ole	Oleuropein
HT	Hydroxytyrosol
OL	Oleacin; Oleuropein-aglycone di-aldehyde
OC	Oleocanthal; (-)-deacetoxy-dialdehydic ligstroside aglycone
